# Human macrophages differentiated in the presence of vitamin D_3_ restrict dengue virus infection and innate responses by downregulating mannose receptor expression

**DOI:** 10.1371/journal.pntd.0005904

**Published:** 2017-10-11

**Authors:** John F. Arboleda Alzate, Izabela A. Rodenhuis-Zybert, Juan C. Hernández, Jolanda M. Smit, Silvio Urcuqui-Inchima

**Affiliations:** 1 Grupo Inmunovirología, Facultad de Medicina, Universidad de Antioquia UdeA, Medellín, Colombia; 2 Department of Medical Microbiology, University of Groningen and University Medical Center Groningen, Groningen, The Netherlands; 3 Infettare, Facultad de Medicina, Universidad Cooperativa de Colombia, Medellín, Colombia; University of North Carolina at Chapel Hill, UNITED STATES

## Abstract

**Background:**

Severe dengue disease is associated with high viral loads and overproduction of pro-inflammatory cytokines, suggesting impairment in the control of dengue virus (DENV) and the mechanisms that regulate cytokine production. Vitamin D_3_ has been described as an important modulator of immune responses to several pathogens. Interestingly, increasing evidence has associated vitamin D with decreased DENV infection and early disease recovery, yet the molecular mechanisms whereby vitamin D reduces DENV infection are not well understood.

**Methods and principal findings:**

Macrophages represent important cell targets for DENV replication and consequently, they are key drivers of dengue disease. In this study we evaluated the effect of vitamin D_3_ on the differentiation of monocyte-derived macrophages (MDM) and their susceptibility and cytokine response to DENV. Our data demonstrate that MDM differentiated in the presence of vitamin D_3_ (D_3_-MDM) restrict DENV infection and moderate the classical inflammatory cytokine response. Mechanistically, vitamin D_3_-driven differentiation led to reduced surface expression of C-type lectins including the mannose receptor (MR, CD206) that is known to act as primary receptor for DENV attachment on macrophages and to trigger of immune signaling. Consequently, DENV bound less efficiently to vitamin D_3_-differentiated macrophages, leading to lower infection. Interestingly, IL-4 enhanced infection was reduced in D_3_-MDM by restriction of MR expression. Moreover, we detected moderate secretion of TNF-α, IL-1β, and IL-10 in D_3_-MDM, likely due to less MR engagement during DENV infection.

**Conclusions/Significance:**

Our findings reveal a molecular mechanism by which vitamin D counteracts DENV infection and progression of severe disease, and indicates its potential relevance as a preventive or therapeutic candidate.

## Author summary

Dengue represents a major worldwide concern for public health. Clinical complications rely on vascular leak of fluids and molecules from the bloodstream that leads to a potentially fatal hemodynamic compromise. Disease progression has been related to poor control of dengue virus (DENV) dissemination and excessive production of pro-inflammatory mediators that affect the endothelial function. Vitamin D has been shown to modulate immune responses and to alleviate dengue disease. Here, we studied how addition of vitamin D during macrophage differentiation modulates the functional features of these cells in the context of DENV infection. We observed that vitamin D reduced susceptibility of these cells to DENV infection and down-regulated the virus-induced cytokine response. This phenotype was attributed to downregulation of MR, a molecule hijacked by the virus to gain entry into the cells and a key receptor of the MR/CLEC5A complex that links binding and immune activation during DENV infection. Our study sheds light on the mechanism by which vitamin D can restrict DENV dissemination and the cytokine response in macrophages, indicating the potential relevance of this hormone as a preventive and therapeutic candidate.

## Introduction

During the last decades, there has been an expansion in the geographic range and incidence of dengue virus (DENV) due to spreading of its mosquito vectors, globalization and the lack of a protective tetravalent dengue vaccine [1–3]. It is estimated that nearly a third of the world population is at risk of infection with an annual incidence of 96 million symptomatic cases and high economic burden in countries where active DENV transmission has been identified [4,5]. Infection with DENV may result in a self-limiting febrile illness known as dengue fever with or without warning signs that can progress to severe dengue. Disease severity is hallmarked by hemodynamic compromises that can lead to organ failure, hypovolemic shock and ultimately death [6]. While only a small percentage of cases evolve to severe dengue, progression and severity of dengue disease can differ depending on eco-epidemiology, host genetic factors, age, and virus virulence [7,8]. Additionally, complex interactions between the host immune response and the virus have been proposed as critical factors contributing to the pathogenesis of the disease [9–12].

In general, upon biting by a dengue-infected mosquito, dermal dendritic cells (DC) and macrophages are the main targets of DENV. In the skin, these cells host viral replication and facilitate further dissemination to peripheral tissues [13,14], therefore becoming key drivers in the regulation of DENV-induced immune responses. To initiate infection, DENV has been shown to interact with C-type lectin receptors such as the mannose receptor (MR), Dendritic Cell-Specific Intercellular adhesion molecule-3-Grabbing Non-integrin (DC-SIGN) and C-type lectin domain family 5 member A (CLEC5A) [15–18]. Although MR and DC-SIGN can bind DENV with high avidity facilitating attachment of the virus to the cell, in macrophages, only MR is thought to play a predominant role in virus binding and signaling [16,18]. Ligation of MR to DENV facilitates spatial interaction of the virus with a lower avidity receptor, CLEC5A [18], which in turn initiates signaling pathways that aim at the secretion of cytokines that potentiate immediate local response and priming of the immune system [17,18].

DENV-induced activation of target cells is generally believed to induce excessive production of pro-inflammatory cytokines such as TNF-α and IL-1β that affect endothelial integrity and consequently enhance capillary permeability [9,10,19,20]. Furthermore, early in the infection, components of mosquito saliva and local tissue damage at the site of infection cause neutrophils, basophils and mast cells to elicit a Th2 cytokine response with predominant production of IL-4 [21]. Notably, the presence of IL-4 induces up-regulation of MR expression on dermal macrophages and recruits monocyte-derived macrophages (MDM), thereby boosting further infection and pro-inflammatory events that may lead to disease progression [13,16,21].

Currently, no treatment for clinical improvement of dengue disease symptoms is available [3,22]; however, antiviral and immunomodulatory factors such as vitamin D may have the necessary potential. Apart from its classical role in maintaining calcium homeostasis, vitamin D_3_ is a potent modulator of the immune system [23]. Indeed, binding of the biologically active form of vitamin D, 1,25-dihidroxyvitamin D_3_ (vitamin D_3_), to the vitamin D receptor (VDR) allows the VDR to act as a transcription factor that modulates the gene expression of proteins involved in calcium absorption, cell proliferation and differentiation [24]. Vitamin D_3_ modulates the immune response to several pathogens including DENV [25–28]. In fact, epidemiological studies have associated genetic variants in the VDR with disease progression and vitamin D supplementation with early disease recovery [29–31]. *In vitro*, vitamin D_3_ treatment of myelo-monocytic cell lines reduces DENV infection and to modulates the cytokine response [32–34]; yet the underlying mechanism remains elusive [35]. Therefore, we here investigated the phenotypic features, susceptibility and innate responses to DENV infection of monocyte-derived macrophages differentiated in the presence of D_3_ (D_3_-MDM).

## Methods

### Ethics statement

Protocols for sample collection and written informed consent were approved by the Committee of bioethics Research of the Sede de Investigación Universitaria, Universidad de Antioquia (Medellín–Colombia).

### DENV stocks and titration

The DENV-2 New Guinea C (NGC) strain was provided by the Center for Disease Control (CDC, CO, USA) and was propagated in C6/36 cells. Briefly, monolayers of C6/36 HT cells in 75-cm^2^ tissue culture flasks were inoculated with DENV at a MOI of 0.05 in 1 mL of L-15 medium supplemented with 2% Fetal Bovine Serum (FBS). After 3 h, 10 mL of L15 medium containing 2% FBS were added and the cells were cultured for 5 days at 34°C without CO_2_. The supernatants were obtained by centrifugation for 5 min at 1800 rpm to remove cellular debris and were stored at -70°C. Virus titration was performed by flow cytometry as described [36]. Briefly, C6/36 HT cells were seeded in 12-well plates and cultured overnight at 34°C without CO_2_. The cells were infected with 10-fold serial dilutions of the virus and harvested at 24 h post-infection (hpi). Indirect intracellular staining of DENV E protein with the monoclonal antibody 4G2 (Millipore, Darmstadt, Germany) and the secondary antibody goat anti-mouse IgG-FITC (Invitrogen, Life Technologies, CA, USA) was performed as described later below. The cells were analyzed by FACScanto flow cytometry using the FACSdiva software. The percentage of infected cells in each sample and the total number of cells seeded per well were used to calculate the final titer of the virus. Isolation of viral RNA from cell lysates and supernatants was performed according to manufacturer’s instructions using the RNeasy mini kit and the QIAamp Viral RNA Mini Kit (Qiagen, Hilden, Germany), respectively. The number of genome equivalent copies (GEc) was determined by RT-qPCR using DENV-2 specific primers (forward: 5’CAATATGCTGAAACGCGAGAGAAA 3’, and reverse: 5’ CCCCATCTATTCAGAATCCCTGCT 3’). The calculation of the GEc was performed based on a standard curve, as previously reported [37,38].

### Cell lines

The mosquito C6/36 HT cell line was obtained from ATCC and cultured in Leibovitz L-15 medium (L-15) supplemented with 10% v/v heat-inactivated FBS, 4 mM L-glutamine, and 10 units/ml penicillin/0.1 mg/ml streptomycin (Sigma-Aldrich Chemical Co, MO, USA), at 34°C in an atmosphere without CO_2_.

### Blood donors

This study was conducted according to the principles expressed in the declaration of Helsinki. All samples of venous peripheral blood were obtained in Medellin-Colombia from an equal proportion of healthy women and men that were not vaccinated against yellow fever virus, were seronegative for the DENV NS1 antigen and DENV IgM/IgG, and were between 20 and 33 years old.

### Monocyte-derived macrophage differentiation

Peripheral blood from donors was mixed with EDTA 4% v/v and human peripheral blood mononuclear cells (PBMCs) were isolated by Ficoll-Histopaque (Sigma-Aldrich) gradient at 650 g during 30 min as described [39]. Platelet depletion was performed by washing with PBS (Sigma-Aldrich) three times at 250 g during 10 min. Monocytes were obtained from total PBMCs by plastic adherence as described [39]. Briefly, 5x10^5^ CD14+ cells from total PBMCs were plated in 24 well plastic plates and were allowed for to adhere during 4 h in 1640 RPMI medium (Sigma-Aldrich) supplemented with 0.5% of heat inactivated human serum pool (HSP) at 37°C and 5% of CO_2_. Non-adherent cells were removed by washing twice with PBS, and MDM differentiation was allowed for 144 h in RPMI medium 10% HSP at 37°C and 5% of CO_2_. Additionally, MDMs were differentiated in presence of 1,25 di-hydroxyvitamin D_3_ to obtain D_3_-MDMs. For this, 1,25 di-hydroxyvitamin D_3_ (Sigma-Aldrich) was added to the culture media at a final concentration of 0.1 nM and replenished every 48 h until the final time point of differentiation was reached. Kinetics and concentration of the vitamin D dose was determined on the basis of cytotoxicity levels lower than 5% (measured by the MTT and trypan blue exclusion assays, of transcriptional induction of vitamin D signaling targets (VDR and CYP24A) and of modulation of immune responses, as previously described [40,41]. For each experiment, two equal fractions of PBMCs were used from the same donor for MDM and D_3_-MDM differentiation. At the indicated experiments, after MDM and D_3_-MDM differentiation, the cells were stimulated with IL-4 (100 ng/mL) (PeProtech) or mock-treated (culture medium) and incubated for an additional 48 h previous to DENV challenge.

### Flow cytometry analysis

Expression of macrophage surface and intracellular molecules was evaluated by flow cytometry. Intracellular staining using anti-CD68-PE (BD Pharmingen) and surface staining using anti-CD14-FITC (BD Pharmingen), CD83-PeCy-7 (eBioscience) and anti-CD206-PE (BD Pharmingen) were performed on MDMs and D_3_-MDMs. For each experiment, unstained and isotype controls were included. DENV infection was measured by intracellular detection of the E protein using the murine monoclonal 4G2 antibody (Millipore, Darmstadt, Germany) and a secondary antibody, the goat anti-mouse IgG-FITC (Invitrogen). Unstained cells, mock-infected cells plus secondary antibody only, mock-infected plus detection pair and infected cells plus secondary antibody were included as controls for every experiment. The percentage of infected cells was expressed as number of 4G2 positive cells over the total number of cells analyzed. Surface and intracellular staining samples were read on a FACScanto flow cytometer (BD Biosciences) and data were analyzed using the FACSdiva (BD Biosciences) and Kaluza (Beckman Coulter) softwares.

### DENV infections

After differentiation, macrophage monolayers were washed with warm PBS and were infected with DENV at the indicated MOI in 300 uL of RPMI medium and 2% HSP per well. In some experiments, macrophage monolayers were pre-treated with α-Methyl D-Mannoside (MM) (Sigma-Aldrich) at 10 mM for 1 h before the virus was added to the cells. For these experiments, mock vehicle controls of cells pre-treated with the same volume of MM diluent alone were included. At 3 hpi, the cells were washed with warm PBS to remove unbound virus and were incubated at 37°C with 5% CO_2_ in RPMI medium and 10% PSH for another 21 h. At 24 hpi, macrophage monolayers were harvested and used for flow cytometry staining and analysis as mentioned above. Additionally, in some experiments cell lysates and supernatants were used to determine the GEc titer.

### DENV binding measurement

Since low temperature can not only affect the dynamics of C-type lectin receptors and the fluidity of the cell membranes to facilitate endocytosis, but also the DENV conformational structure and attachment to target cells [42,43], we conducted our binding experiments at 37°C, as previously described [44]. Macrophages were infected with DENV at a MOI of 10 for 1 h. Thereafter the cells were delicately washed twice with PBS to remove the unbound virus and lysed to determine the GEc titers as described above. Since rapid internalization may occur during this time, we report here percentage of bound and/or internalized GEc. The number of GEc titers detected in the inoculum served as 100% binding and/or internalization control.

### Gene expression measurement

mRNA expression of some macrophage genes was measured by quantitative real-time PCR. For total RNA isolation, an RNeasy Mini Isolation Kit (Qiagen, Valencia, CA, USA) was used. For cDNA synthesis, the RevertAid Minus First Strand cDNA Synthesis Kit (Thermo Scientific, Wilmington, DE, USA) was used according to the manufacturer’s instructions. Quantitative real-time PCR reactions were performed using the following primers. For CD206 (Mannose receptor), forward: 5’-CACGATCCGACCCTTCCTTG-3’ and reverse: 5’GCTTGCAGTATGTCTCCGCT-3’. For CLEC5A forward: 5’-CTTCCAGGGAGAAAGAGGCCC-3’ and reverse: 5’-CTGGTGGTGGTGAAACCATCG-3’. For CD209 (DC-SIGN) forward: 5’-GAGTTCTGGACACTGGGGGAG-3 and reverse: 5’-CAAGACACCCTGCTAAGCTCTTG-3’. For CYP24A1, forward 5'-ACCAGGGGAAGTGATGAAGC-3' and reverse 5'-GTACAAGTCTTCAACGTGGC-3'. For VDR, forward: 5’-TGCTATGACCTGTGAAGGCTG-3’ and reverse: 5’-AGTGGCGTCGGTTGTCCTT-3’. For β2M, forward 5'-GAGTATGCCTGCCGTGTG-3’ and reverse 5’-AATCCAAATGCGGCATCT-3’. The Bio-Rad CFX manager was used to obtain the cycle thresholds (Ct) that were determined in each sample using a regression fit in the linear phase of the PCR amplification curve. Duplicate assays were performed for each sample and relative transcript units (RTU) and fold-change values were calculated in relation to β2M (define) expression by using the ΔCt and the ΔΔCt method, respectively.

### Cytokine response

Levels of TNF-α, IL-6, IL-1β and IL-10 in cell supernatants were measured by ELISA (BD Biosciences, San Jose, CA, USA) according to the manufacturer’s instructions.

### Statistical analysis

Statistical comparisons were performed using the non-parametric Mann-Whitney test using the software GraphPad Prism 5 (GraphPad Prism, CA, USA). In addition, the Wilcoxon signed-rank test was used to compare paired MDMs and D_3_-MDM data. The critical value for statistical significance used for the analysis in the present study was *p*<0.05, denoted as *, p<0.01 denoted as **, and p<0.001 denoted as ***.

## Results

### D_3_-MDMs are less susceptible to DENV infection than MDMs

First, we assessed the effect of D_3_-MDM differentiation on the main phenotypic features of MDMs. To this end, CD14+ monocytes were obtained from human PBMCs by plastic adherence as described [39], and were cultured during 144 h in the absence (MDM) or presence of 0.1nM vitamin D_3_ (D_3_-MDM). Cell yield, viability and morphology were not affected by the presence of vitamin D_3_ during MDM differentiation (S1A Fig). Likewise, both MDMs and D_3_-MDMs were positive for CD68 and showed a similar low expression of CD83 (S1B Fig). As expected, D_3_-MDM differentiation induced an increase in the mRNA levels of two vitamin D inducible target genes, the VDR and hydroxylase CYP24A [45] (S1C Fig). This confirmed the functional induction of vitamin D_3_ signaling in D_3_-MDMs.

**Fig 1:**
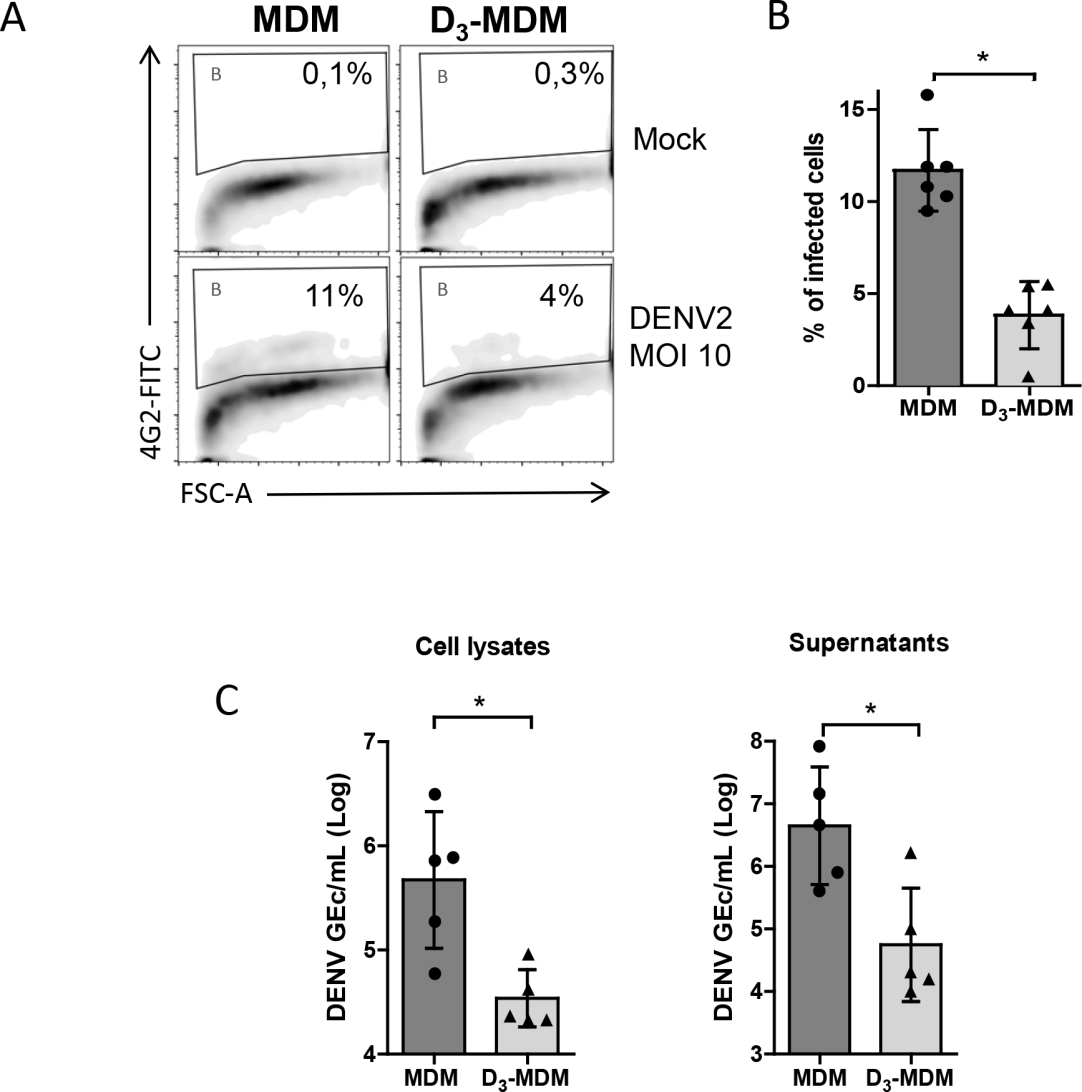
Susceptibility of MDMs and D_3_-MDMs to DENV-2 infection. After differentiation, the cells were challenged with DENV-2 at a MOI of 10 and 24 hpi cells, cell lysates and supernatants were obtained. **A**. FACS measurement of DENV infection as number of positive cells for intracellular DENV-E protein. **B.** DENV infection measurement and comparison between MDMs and D_3_-MDMs at a MOI of 10. **C.** GEc titers measured by RT-qPCR in cell lysates and supernatants. Bars represent mean ± SD. Wilcoxon signed rank test; *p<0.05, **p<0.01.

Next, we compared the susceptibility of MDMs and D_3_-MDMs towards the DENV-2 NGC strain. Infection was evaluated 24 hpi by intracellular detection of the viral E glycoprotein using flow cytometry. In line with an earlier publication [34], we observed that DENV-2 infection in MDMs depends on the MOI. At a MOI of 10, 9.5% of the cells were infected (S1D Fig and Fig 1A). Importantly, and in line with studies in immortal cells, infection of D_3_-MDMs at a MOI of 10 resulted in a significantly lower (*p* = 0.01) percentage of DENV-positive cells (~4%) (Fig 1A and 1B). To verify these results, we measured the number of DENV (GEc present in cell lysates and supernatants. In line with the infection data, we found that both, intracellular and secreted GEc titers were significantly lower (*p* = 0.03) in D_3_-MDMs when compared to MDMs (Fig 1C). Interestingly, whereas the percentage of infected cells was reduced only by 2 fold, the viral progeny release was reduced by 200 fold, suggesting that D_3_-MDM differentiation may contribute to viral restriction during early and late stages of the viral life cycle.

### DENV-2 binds less efficiently to D_3_-MDMs

**Fig 2:**
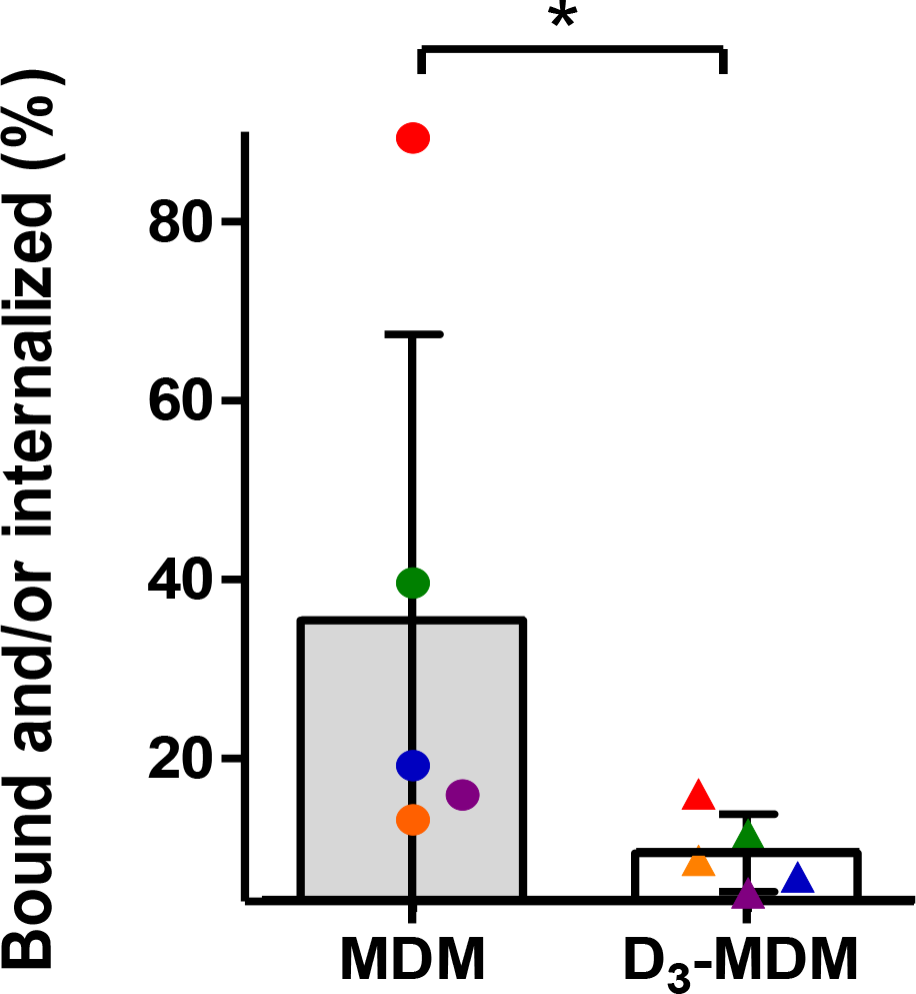
Binding and/or internalization of DENV into MDMs and D_3_-MDMs. The bound and/or intracellular virus particles were detected using RT-qPCR as described in the Methods section. The percentage of bound-internalized viral particles was calculated in relation to the GEc added (equivalent of a MOI of 10). Bars represent mean ± SD; n = 5 Wilcoxon signed rank test *p<0.05.

To assess whether DENV infectivity in D_3_-MDMs is restricted at early stages of infection, we evaluated the number of bound and/or internalized DENV-2 particles in MDMs and D_3_-MDMs at 1 hpi. The number of bound and/or internalized DENV-2 GEc was measured by RT-qPCR as described in the Methods section. Fig 2 shows the percentage of bound and/or internalized DENV-2 GEc in relation to the number of added GEc titers. For MDMs, the percentage of bound and/or internalized DENV-2 GEc ranged from 15% to 80% depending on the blood donors. Notably, only for D_3_-MDMs, was the percentage of bound and/or internalized DENV-2 GEc comparable between the donors and significantly (*p* = 0.03) lower in comparison to their MDM counterparts. This led us to propose that macrophage differentiation in the presence of vitamin D_3_ reduces expression of receptors required for DENV to gain entry into macrophages.

### D_3_-MDM modulates the expression of the mannose receptor

**Fig 3:**
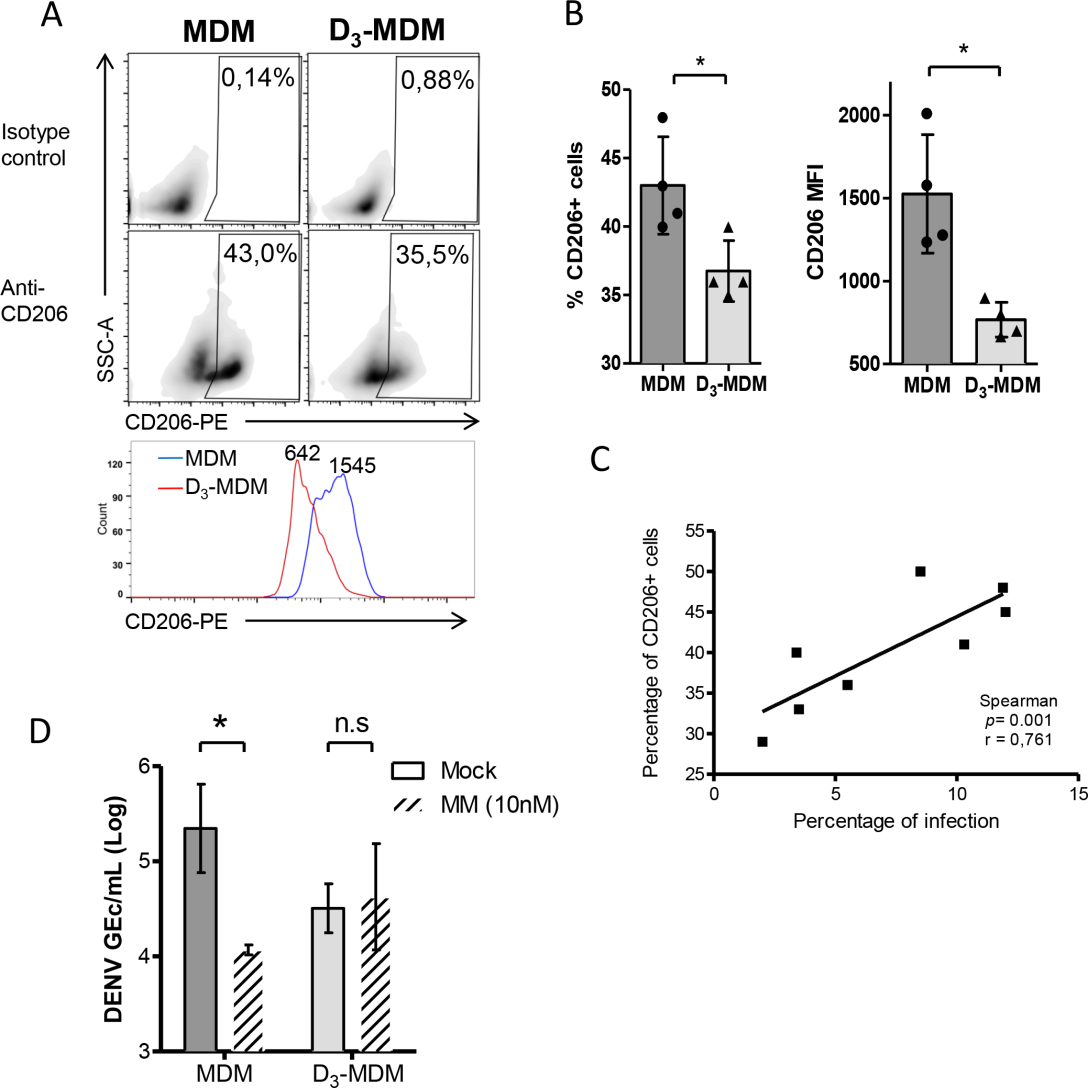
Surface MR expression and effect of its blockade on DENV replication in MDMs and D_3_-MDMs. MR surface expression was measured by FACS detection of CD206+ cells after MDM and D_3_-MDM differentiation. **A.** Flow cytometry-gating strategy for the measurement of CD206+ events from the parental region A from S1B Fig. Isotype controls were used for both MDMs and D_3_-MDMs to set the CD206 positive events gate. Middle and lower panels show representative distribution of CD206+ cells in MDM and D_3_-MDM in gate B and the comparison of CD206 Mean Fluorescence Intensity (MFI). **B**. Statistical comparison of CD206+ percentage cells and CD206 MFI. **C.** Correlation between the percentage of CD206 positive cells and infection percentage in MDMs and D_3_-MDMs observed in 4 different donors. **D.** MR ligation to DENV-2 was blocked by incubating with methyl mannoside (MM) for 2 h prior to infection. The intracellular number of GEc was measured by RT-qPCR 24 hpi and was compared with that in control mock-treated cells. Bars represent mean ± SD from at least 3 different donors. Wilcoxon signed rank test; *p<0.05; n.s denotes non-significant.

Among several C-type lectins expressed on macrophages that can facilitate virus attachment, MR is thought to represent a key binding receptor [15,16,18,46]. Thus, we next tested whether the decreased binding in D_3_-MDMs was due to the limited accessibility of MR on these cells. To this end, surface expression of MR by FACS detection of CD206 in MDMs and D_3_-MDMs was evaluated. As shown in Fig 3A, both MDMs and D_3_-MDMs stained positive for CD206 in all 4 donors tested. Yet, the percentage of CD206 positive cells was significantly lower (*p* = 0.04) in D_3_-MDMs (~36%) when compared with MDMs (~43%) for all donors (Fig 2B). In addition, the mean fluorescence intensity (MFI) values of CD206 were lower in D_3_-MDMs (~750) as compared with those observed in MDMs (~1400) (Fig 3A and 3B). Interestingly, we observed a positive correlation between the percentage of CD206 positive cells and the percentage of infection in MDMs and D_3_-MDMs (*p* = 0.001, r = 0.761) (Fig 3C). Moreover, and in line with our flow cytometry data, we also found that the transcriptional activity of MR was significantly (*p* = 0.01) lower in D_3_-MDMs than in MDMs (S2A Fig), suggesting an overall reduction in MR availability. Likewise, the same pattern of transcriptional activity was observed for CLEC5A (S2B Fig), however, for DC-SIGN, it was not detected in either MDMs or D_3_-MDMs (S2C Fig). This is in line with previous reports where transcriptional and surface expression of DC-SIGN has not been detected in MDMs obtained under the same conditions as reported here [47]. Since MR is critical for the MR/CLEC5A complex function, the reduced susceptibility of D_3_-MDMs to DENV could be attributed to the lower expression of MR on the cell surface.

To confirm this result, MR and other C-type lectins were blocked, reasoning that this treatment would have no effect on DENV infection in D_3_-MDMs. Accordingly, cells were pre-treated with α-Methyl-D-mannoside (MM), a mannose binding site competitor and inhibitory sugar of C-type lectins [48]. As shown in Fig 3D, pre-treatment of MDMs with 10 mM MM reduced the intracellular DENV-2 GEc titers by approximately ~1 Log as compared with those observed in mock pre-treated control cells. Of note, the intracellular GEc titers in MDMs treated with MM were virtually identical to those found in D_3_-MDMs. Importantly, MM treatment had no effect on the GEc titers observed in D_3_-MDMs thereby confirming that low accessibility of C-type lectins indeed restricts infection of D_3_-MDMs.

### D_3_-MDMs attenuate IL-4-induced susceptibility to DENV

**Fig 4:**
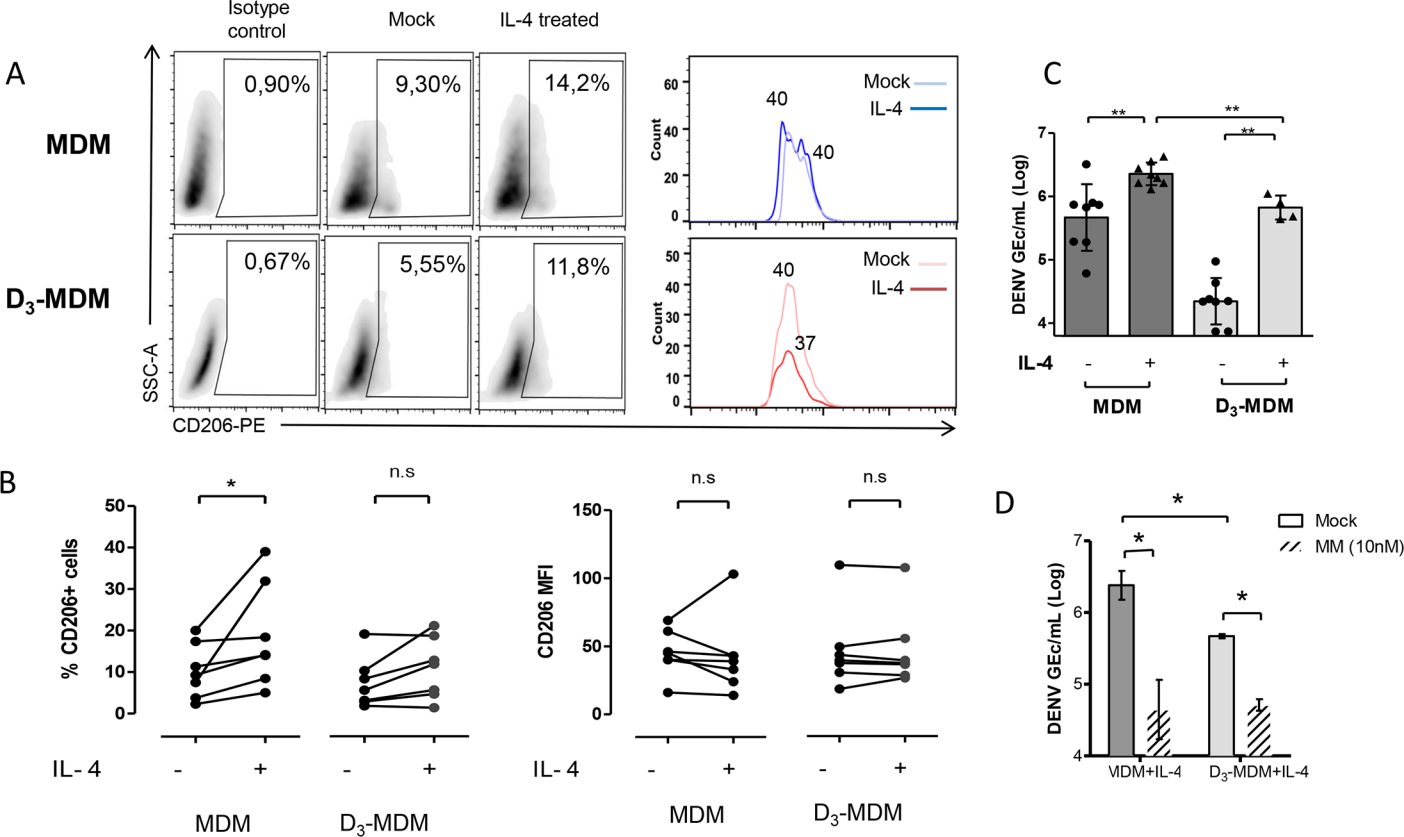
IL-4 induced MR expression and DENV infection in MDMs and D_3_-MDMs. After MDM and D_3_-MDM differentiation, the cells were stimulated with IL-4 and MR induction was allowed for an additional 48 h. **A.** Surface detection of CD206 was measured by flow cytometry. Isotype controls were used for both MDMs and D_3_-MDMs. Dot plots show representative distribution of CD206+ cells in MDMs and D_3_-MDMs with and without IL-4 treatment. Histograms show the comparison of the CD206 MFI. **B**. Statistical comparison of CD206 MFI and percentage of CD206+ cells for all 5 donors tested. **C.** IL-4 treated MDMs and D_3_-MDMs were infected with DENV and 24 hpi, the numbers of virus genome particles were measured by RT-qPCR in cell lysates and compared with those in mock-treated cells. **D**. MR ligation to DENV-2 was blocked with MM 2 h prior to infection. The intracellular numbers of GEc were measured by RT-qPCR 24 hpi, and compared with those in control mock-treated cells. Bars represent mean ± SD from at least 3 different donors. Wilcoxon signed rank test and Mann-Whitney test. *p<0.05, **p<0.01, *** *p*<0,001. n.s = not significant.

To substantiate the contribution of limited MR accessibility to reduced infection in D_3_-MDMs, we sought to up-regulate MR expression by treatment with IL-4, a well-known inducer of MR expression in macrophages [16,46]. To this end, MDMs and D_3_-MDMs were treated with IL-4 (100 ng/mL) for 48 h, as described [16]. Analysis of MR expression by flow cytometry showed that IL-4 treatment induced a significant (*p* = 0.007) increase in the percentage of MR positive cells in MDMs but not in D_3_-MDMs (Fig 4A and 4B). In addition, no differences were observed in the MFI values of CD206 positive cells. This indicates that D_3_-MDMs are refractory to the canonical effects of IL-4 on MR expression. In line with this, we observed that in MDMs, IL-4 treatment induced an 8-fold increase in the transcriptional activity of the MR gene when compared to mock-treated, whereas in IL-4-treated D_3_-MDMs, the fold-change induction was only 2-fold (S2D Fig). Likewise, induction of CLEC5A transcriptional activity was lower in D_3_-MDMs when compared with MDMs (S2E Fig), indicating that other IL-4-induced c-type lectins may also be less responsive to the effect of vitamin D.

We then tested whether this limited IL-4-induced increase of MR expression in D_3_-MDMs was also linked to reduced susceptibility to DENV-2. Thus, IL-4-treated MDMs and D_3_-MDMs were infected with DENV-2 at a MOI of 10 and 24 hpi the number of DENV GEc was determined by RT-qPCR in cell lysates (Fig 4C). IL-4 treatment increased the susceptibility of both MDMs and D_3_-MDMs as compared with their control, the mock-treated cells. As expected, for the mock-treated control D_3_-MDMs, the number of DENV GEc titers were ~1.5 log lower as compared with mock-treated control MDMs. However, the number of intracellular DENV GEc observed in IL-4-treated D_3_-MDMs was significantly lower than that found in IL-4-treated MDMs (*p* = 0.007). In spite of this, there was no detectable increase in MR expression in D_3_-MDMs, IL-4 did increase the susceptibility to DENV infection. This rather surprising finding prompted us to investigate the role of MM in controlling DENV infectivity in IL-4-treated cells. To this end, the number of DENV GEc was determined in IL-4-treated cells at 24 hpi (Fig 4D). MM treatment lowered GEc production by ~2 Log (*p* = 0.01) and ~1 Log (*p* = 0.01) in MDMs and D_3_-MDMs, respectively, as compared with mock-treated control cells. All these data suggest the importance of MR during DENV infection of MDMs and D_3_-MDMs and provide insights of in the mechanism by which vitamin D limits DENV infection in human macrophages.

### DENV-induced inflammatory response is diminished in D_3_-MDMs

**Fig 5:**
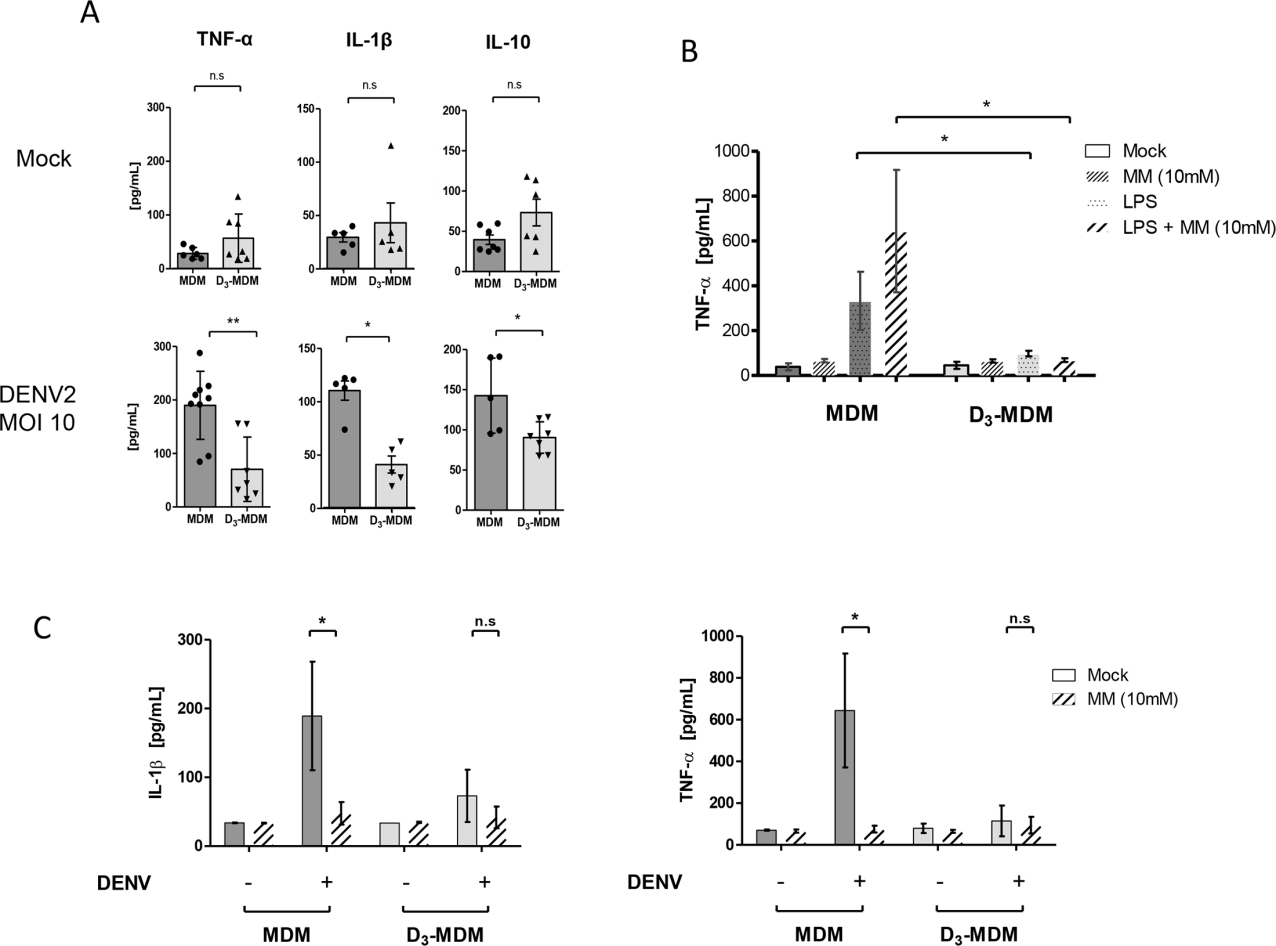
DENV-induced cytokine response in MDMs and D_3_-MDMs. **A**. Levels of cytokines released by the two macrophage preparations following DENV infection at 24 hpi. **B** Effect of 10mM MM treatment on the LPS (10 ng/mL)-induced TNF-α secretion in MDMs and D_3_-MDMs. **C.** DENV-induced secretion of TNF-α and IL-1β after MM treatment in MDMs and D_3_-MDMs. Cells were pre-incubated with 10 nM MM prior to infection with DENV. Bars represent mean ± SD. Wilcoxon signed rank test. *p<0.05, **p<0.01, ***. n.s denotes not significant.

Besides the role of macrophages as target cells for DENV infection, they also play an important role in the production of pro-inflammatory cytokines that can enhance the pathogenesis of dengue disease [12]. Since vitamin D_3_ has been previously suggested to act as an important modulator of immune responses [41], we next evaluated changes in cytokine response of MDMs and D_3_-MDMs following DENV infection. To this end, we measured by ELISA in culture supernatants, the production of several cytokines related to DENV pathogenesis, including TNF-α, IL-1β and IL-10. As shown in Fig 5A, baseline levels (mock-infected cells) of the cytokines are similar between MDMs and D_3_-MDMs. However, upon DENV infection, the induced cytokine levels were significantly lower (*p*<0.01 and *p*<0.05) in D_3_-MDMs as compared with MDMs (Fig 5A). This observation could be a direct consequence of the lower infection or poor engagement of C-type lectin receptors, such as MR, due to the overall down-regulation of these molecules observed in D_3_-MDMs.

DENV attachment to MR allows spatial interaction and cooperation of the MR/DENV complex with CLEC5A, that interacts with the virus, and triggers the DENV-induced pro-inflammatory signaling pathways [18]. Since we observed down-regulation of MR and CLEC5A in D_3_-MDMs, we hypothesized reduced participation of these receptors during DENV-induced cytokine response in these cells. Accordingly, we evaluated the DENV-induced cytokine response after blockade of C-type lectin ligation to DENV using MM. As shown in Fig 5B, pre-treatment of the cells with 10 nM MM had no effect on the levels of TNF-α, as compared with mock-treated control cells. The MM pretreatment also did not affect TLR4/CD14-mediated signaling indicating that incubation with MM does not interfere with the secretion of pro-inflammatory mediators induced by C-type lectin receptor-independent pathways. Of note, and in line with previous reports [32,41], LPS did not induce TNF-α production in D_3_-MDMs, substantiating the reduction of inflammatory responses in D_3_-MDMs. Additionally, MM treatment itself did not alter the secretion of TNF-α and IL-1β in MDMs and D_3_-MDMs. Importantly, upon DENV infection, MM treatment reduced the secretion of TNF-α and IL-1β in MDMs but not in D_3_-MDMs. On the other hand, in D_3_-MDMs, these cytokine levels were significantly (p<0.05) lower than in MDMs and showed the same levels as those found in MM-treated cells. Taken together, these data suggest that in MDMs and D_3_-MDMs, C-type lectin ligation to DENV triggers cytokine release and supports the hypothesis that down-regulation of these molecules can contribute in modulating the DENV-induced cytokine response in D_3_-MDMs.

## Discussion

Containment of DENV infection and controlled secretion of inflammatory cytokines by macrophages are crucial events needed to avoid progression of dengue disease [12,49]. Our findings show that differentiation in the presence of vitamin D_3_ restricts DENV infection in human MDMs by affecting DENV binding to cells. We found that MR is reduced in D_3_-MDMs and given the importance of this receptor for DENV attachment, we argue its accessibility as a limiting factor for less virus binding. Although we cannot rule out the participation of other C-type lectins such as CLEC5A, engagement of the MR receptor during DENV infection depends on the formation of the MR-DENV complex [18], attributing to MR an essential role for binding and signaling. Indeed, we show that reduced expression of MR and likely of CLEC5A contributes to a lower secretion of DENV-induced TNF-α, IL-1β and IL-10.

The expression of MR is under the control of the pro-adipogenic peroxisome proliferator-activated receptor γ (PPARγ) [50], a transcription factor that is responsible for the induction of MR expression via IL-4/IL-13 signaling and polarization of inflammatory responses in macrophages [51]. Interestingly, vitamin D activity was recently found to downregulate PPARγ expression and co-regulate PPARγ-induced transcriptional activity in macrophages [52,53]. This could explain the reduced expression of MR in D_3_-MDMs and a limited effect of IL-4 on these macrophages. Importantly, this may also have *in vivo* relevance in the context of dengue pathogenesis, as since inflammatory skin conditions caused by mosquito bites are accompanied by secretion of IL-4 that leads to enhancement of infection and cytokine response [16,46]. It is important to note, that the effects of vitamin D on MR expression reported here may contrast previous reports [54,55], where vitamin D_3_ concentrations and target cells were different to from those used in the present study.

Furthermore, we showed here that secretion of TNF-α, IL-1β and IL-10 was significantly lower in DENV-infected D_3_-MDMs than in MDMs. This observation is in line with a previous report in the human cell line U937 [56] and can be a direct consequence of either lower cell activation due to the decreased viral load and less availability of viral antigens, or to the immunomodulatory features of vitamin D_3_. In the latter scenario, it is recognized that vitamin D can control cytokine responses by indirect modulation of NF-κB activity or by direct regulation of VDR-dependent genes [32,57–59]. Both mechanisms may contribute to variations in the expression of several PRRs that are important to trigger cytokine responses. Indeed, our data shows that MDM differentiation in the presence of vitamin D modulates several DENV-relevant PRRs, such as MR and CLEC5A. MR orchestrates the MR/DENV/CLEC5A complex functions to provide an essential connection between binding and triggering of downstream signaling pathways that aim at cytokine secretion [18]. Since we found diminished MR expression in D_3_-MDMs, it is likely that a lower participation of the MR/CLEC5A complex during DENV infection can occur. Certainly, blockade of MR ligation to DENV in MDMs decreased secretion of TNF-α and IL-1β to the same levels as observed in D_3_-MDMs, showing the effect of MR availability in the induction of cytokine responses during DENV infection.

This study provides a mechanism underlying the resistant phenotype of human MDMs differentiated in the presence of vitamin D_3_ to DENV infection *in vitro*. Interestingly, we also demonstrated that the anti-DENV effect provided by vitamin D_3_ was retained after IL-4 stimulation. Since this cytokine can enhance C-type lectin receptor-mediated-DENV infection and the cytokine response [46,60], our observations indicate a potential *in vivo* role of vitamin D_3_ in down-tuning the immune response during infection by DENV. Interestingly, susceptibility to severe dengue disease has been associated with variations on in VDR and Fc receptors [30], that are crucial for antibody-dependent enhancement of infection during secondary encounters with the virus. However, future and critical studies are required to assess the clinical importance of our findings and the potential role of vitamin D as a preventive or therapeutic target to treat disease severity. Interestingly, several reports have already anticipated VDR genetic variants with clinical outcomes of dengue disease and oral vitamin D supplementation with disease recovery and moderate inflammation [29–31].

## Supporting information

S1 Fig Main phenotypic features of MDM and D_3_-MDM. Monocyte-derived macrophages were differentiated in absence and presence of 1,25-dihidroxyvitamin D_3_ (0.1nM) during 144 h. **A**. Upper panel shows a representative micro-photography of typical “rounded and spindle” morphology in MDM and D_3_-MDM cells. Lower panel shows Forward light scatter versus side light scatter plot and parental gating region in MDM and D_3_-MDM. **B**. Representative MFI histograms for expression of the macrophage marker CD68 and the dendritic cell marker CD83 in MDM and D_3_-MDM. **C.** mRNA expression of Vitamin D related targets in MDM and D_3_-MDM measured by RT-qPCR. mRNA levels are expressed as transcript units relative to β2-microglobulin (RTU). **D** Percentage of DENV E–positive macrophages at 24 hpi at the indicated MOI. Left panel shows representative dot plots for assay controls: unstained cells; Mock infected cells + detection pair of antibodies; Mock infected + secondary antibody and DENV infected cells + secondary antibody. Data from a representative donor (out of at least 3) are shown. Bars represent mean ± SD. Mann-Whitney test; *p<0.05.
(TIF)

S2 Fig mRNA levels of relevant C-type lectins for DENV infection and cytokine profile in MDM and D_3_-MDM. Monocyte-derived macrophages were differentiated in absence and presence of 1,25-dihidroxyvitamin D_3_ (0.1nM) during 144 h and RT-qPCR was performed to determine the transcriptional activity of **A** Mannose receptor (CD206) and **B** CLEC5A and **C** DC-SIGN. Figures **D** and **E** show the fold-change induction of these molecules after treatment with IL-4. Data representative of experiments with at least 4 different donors. Bars represent mean ± SD. Mann-Whitney test; *p<0.05.
(TIF)
