# Supplementary figures and images for: Human macrophages differentiated in the presence of vitamin D3 restrict dengue virus infection and innate responses by downregulating mannose receptor expression
Source: PLoS Negl Trop Dis. 2017 Oct 11;11(10):e0005904. doi: 10.1371/journal.pntd.0005904 (PMC5653353; doi:10.1371/journal.pntd.0005904)

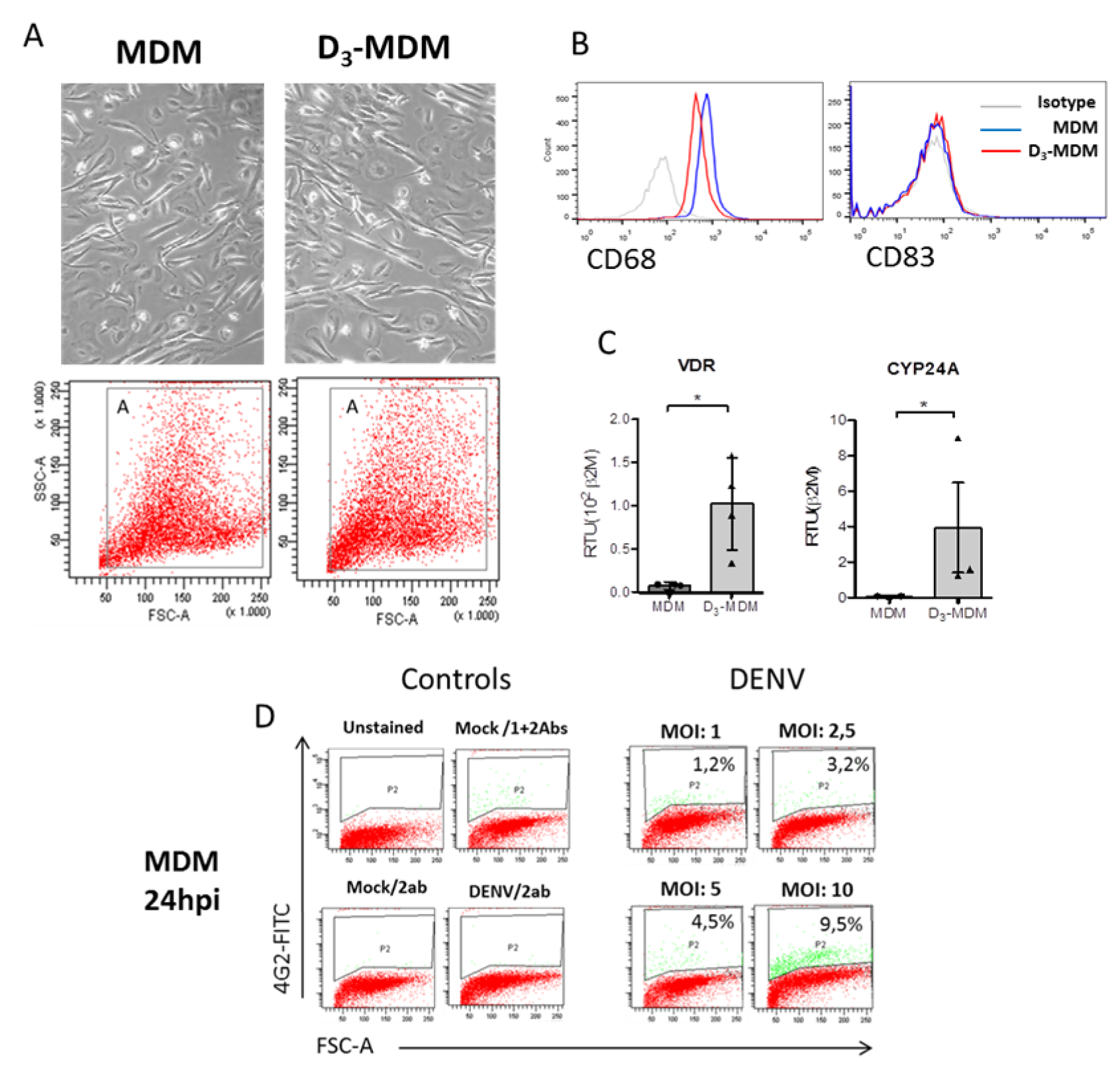

Supplement: S1 Fig — Monocyte-derived macrophages were differentiated in absence and presence of 1,25-dihidroxyvitamin D3 (0.1nM) during 144 h. A. Upper panel shows a representative micro-photography of typical “rounded and spindle” morphology in MDM and D3-MDM cells. Lower panel shows Forward light scatter versus side light scatter plot and parental gating region in MDM and D3-MDM. B. Representative MFI histograms for expression of the macrophage marker CD68 and the dendritic cell marker CD83 in MDM and D3-MDM. C. mRNA expression of Vitamin D related targets in MDM and D3-MDM measured by RT-qPCR. mRNA levels are expressed as transcript units relative to β2-microglobulin (RTU). D Percentage of DENV E–positive macrophages at 24 hpi at the indicated MOI. Left panel shows representative dot plots for assay controls: unstained cells; Mock infected cells + detection pair of antibodies; Mock infected + secondary antibody and DENV infected cells + secondary antibody. Data from a representative donor (out of at least 3) are shown. Bars represent mean ± SD. Mann-Whitney test; *p<0.05. (TIF) [file pntd.0005904.s001.tif]

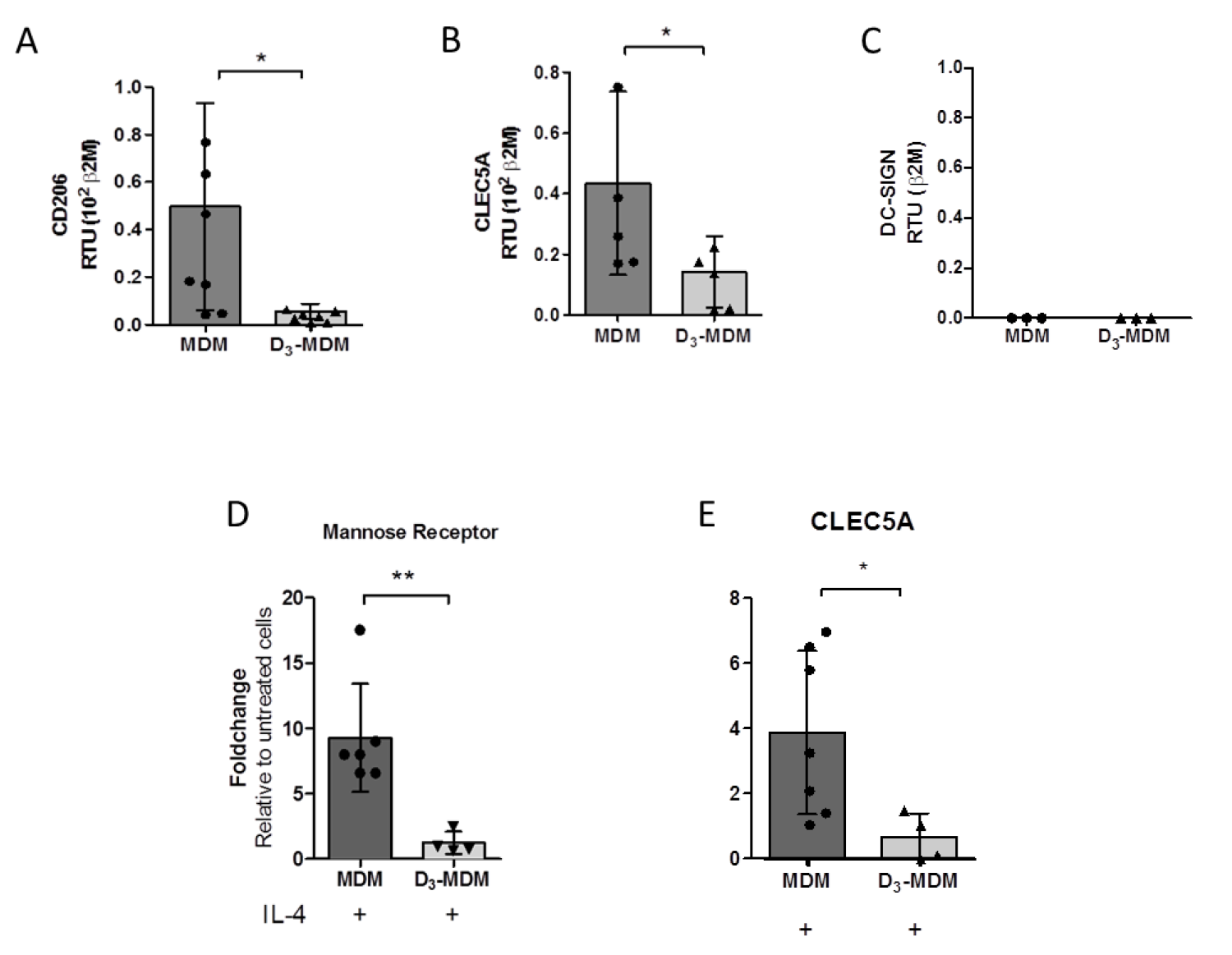

Supplement: S2 Fig — Monocyte-derived macrophages were differentiated in absence and presence of 1,25-dihidroxyvitamin D3 (0.1nM) during 144 h and RT-qPCR was performed to determine the transcriptional activity of A Mannose receptor (CD206) and B CLEC5A and C DC-SIGN. Figures D and E show the fold-change induction of these molecules after treatment with IL-4. Data representative of experiments with at least 4 different donors. Bars represent mean ± SD. Mann-Whitney test; *p<0.05. (TIF) [file pntd.0005904.s002.tif]
